# Author Correction: Layer-by-layer phase transformation in Ti_3_O_5_ revealed by machine-learning molecular dynamics simulations

**DOI:** 10.1038/s41467-025-56264-4

**Published:** 2025-01-23

**Authors:** Mingfeng Liu, Jiantao Wang, Junwei Hu, Peitao Liu, Haiyang Niu, Xuexi Yan, Jiangxu Li, Haile Yan, Bo Yang, Yan Sun, Chunlin Chen, Georg Kresse, Liang Zuo, Xing-Qiu Chen

**Affiliations:** 1 Shenyang National Laboratory for Materials Science, Institute of Metal Research, Chinese Academy of Sciences, Shenyang, 110016 China; 2 School of Materials Science and Engineering, University of Science and Technology of China, Shenyang, 110016 China; 3 State Key Laboratory of Solidification Processing, International Center for Materials Discovery, School of Materials Science and Engineering, Northwestern Polytechnical University, Xi’an, 710072 China; 4 Key Laboratory for Anisotropy and Texture of Materials (Ministry of Education), School of Materials Science and Engineering, Northeastern University, Shenyang, 110819 China; 5 University of Vienna, Faculty of Physics and Center for Computational Materials Science, Kolingasse 14-16, A-1090 Vienna, Austria

**Keywords:** Phase transitions and critical phenomena, Atomistic models

Correction to: *Nature Communications* 10.1038/s41467-024-47422-1, published online 09 April 2024

In the Acknowledgements section of this article the grant number relating to National Natural Science Foundation of China given for J.H. and H.N. was incorrectly given as 22003050 and should have been 92370118. The original article has been corrected.

