# Supplementary material for: Layer-by-layer phase transformation in Ti3O5 revealed by machine-learning molecular dynamics simulations
Source: Nat Commun. 2024 Apr 9;15:3079. doi: 10.1038/s41467-024-47422-1 (PMC11004112; doi:10.1038/s41467-024-47422-1)
Supplement: Supplementary file 1 — Supplementary Information [file 41467_2024_47422_MOESM1_ESM.pdf]

Supplementary Information to "Layer-by-layer phase transformation in Ti_3O_5 revealed by machine learning molecular dynamics simulations"

Mingfeng Liu,[1,2,⋆] Jiantao Wang,[1,2,⋆] Junwei Hu,[3,⋆] Peitao Liu,[1,†] Haiyang Niu,[3,†] Xuexi Yan,[1] Jiangxu Li,[1] Haile Yan,[4] Bo Yang,[4] Yan Sun,[1] Chunlin Chen,[1] Georg Kresse,[5] Liang Zuo,[4], and Xing-Qiu Chen[1]

[1] *Shenyang National Laboratory for Materials Sciences, Institute of Metal Research, Chinese Academy of Sciences, Shenyang 110016, China*
[2] *School of Materials Science and Engineering, University of Science and Technology of China, Shenyang 110016, China*
[3] *State Key Laboratory of Solidification Processing, International Center for Materials Discovery, School of Materials Science and Engineering, Northwestern Polytechnical University, Xi'an 710072, China*
[4] *Key Laboratory for Anisotropy and Texture of Materials (Ministry of Education), School of Materials Science and Engineering, Northeastern University, Shenyang 110819, China*
[5] *University of Vienna, Faculty of Physics, Computational Materials Physics, Kolingasse 14-16, 1090 Vienna, Austria*

† *Corresponding to: ptliu@imr.ac.cn, haiyang.niu@nwpu.edu.cn*
⋆ *These authors contribute equally.*

Supplementary Figures

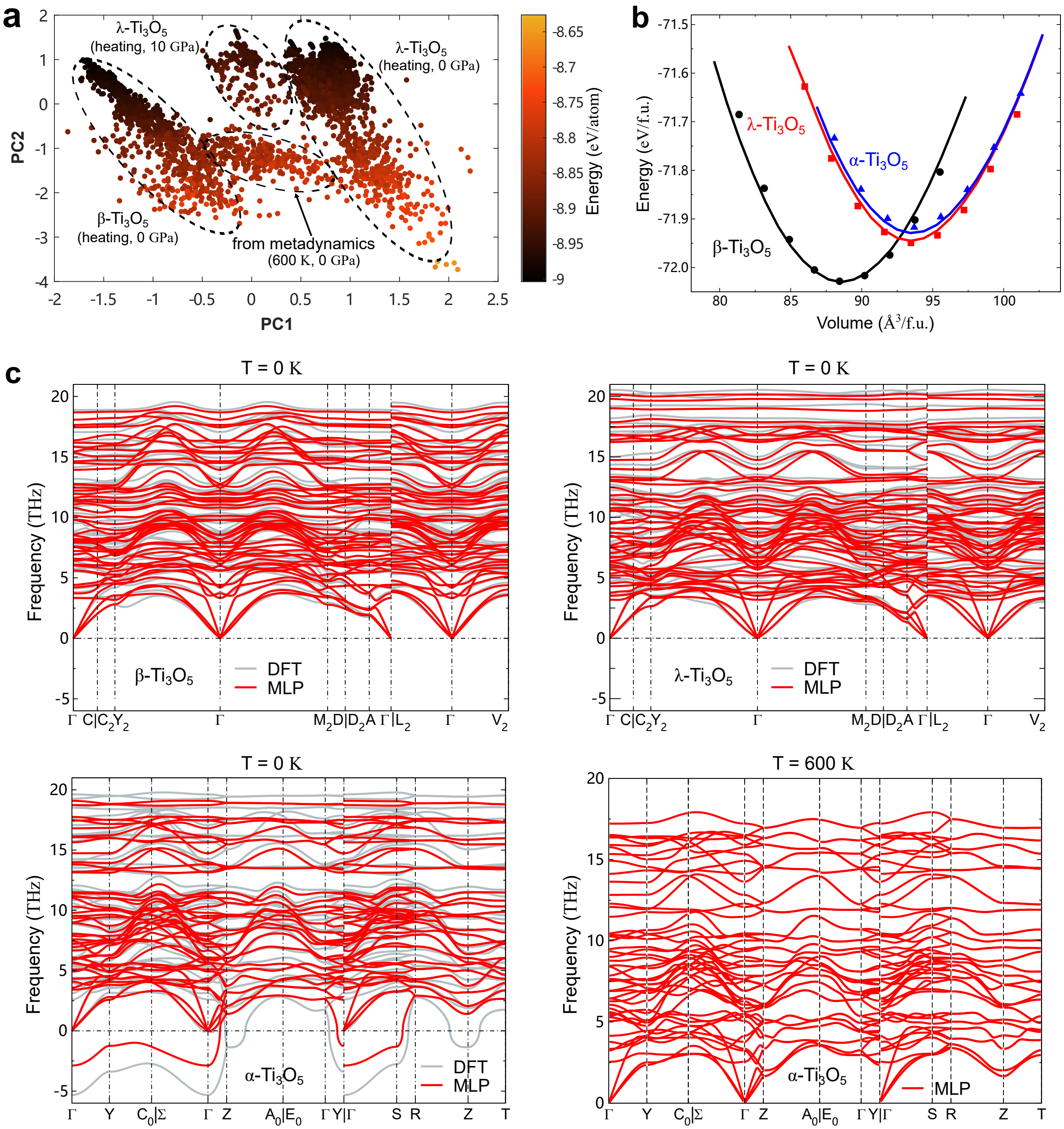

Supplementary Fig. 1. Machine learning potential (MLP) training and validation. a, The kernel principal component analysis map of the entire training structures. The map is color-coded according to the energy per atom. **b**, Energy-volume curves predicted by DFT (circles and squares) and MLP (solid lines). The energy is given in electron volt per formula unit (eV/f.u.). **c**, Phonon dispersion relationships predicted by DFT (gray lines) and MLP (red lines).

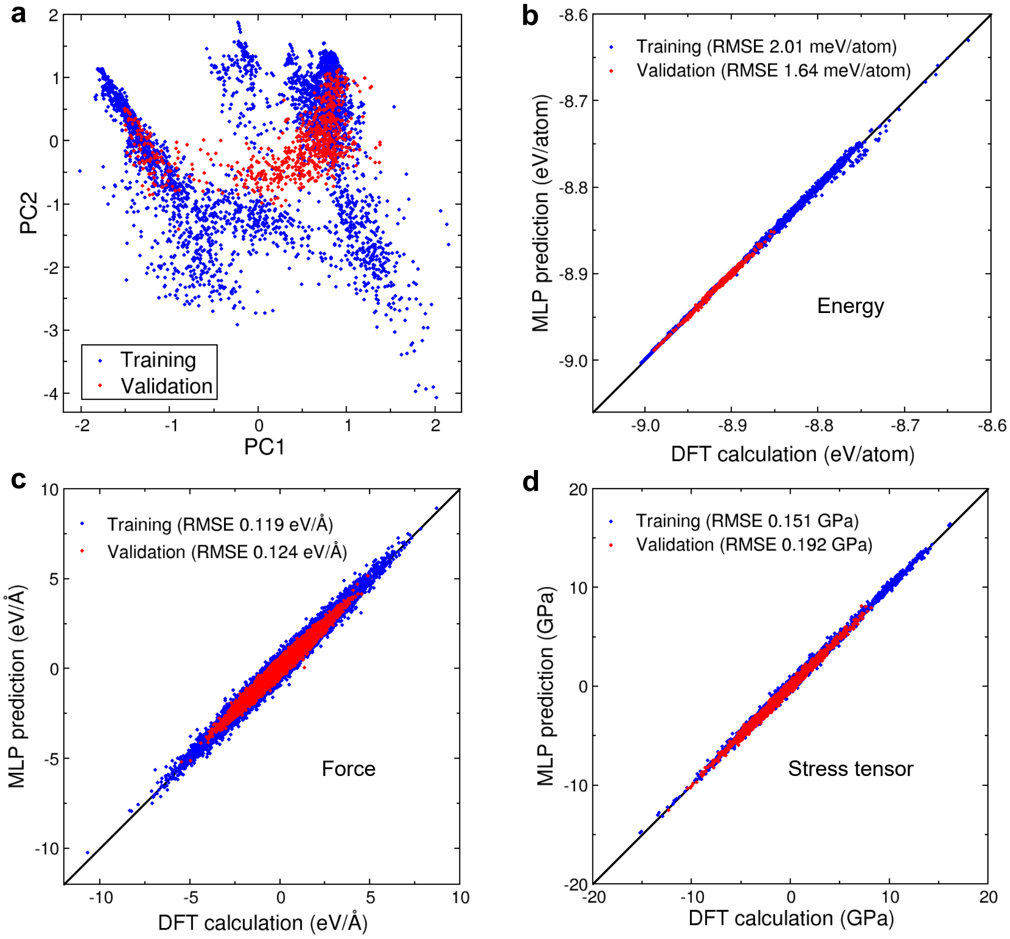

Supplementary Fig. 2. Training and validation datasets and machine learning potential (MLP) predictions vs. DFT results. a, The kernel principal component analysis map of training structures (blue squares) and validation structures (red squares). **b**, MLP predicted energies vs. DFT results. **c**, MLP predicted forces vs. DFT results. **d**, MLP predicted stress tensors vs. DFT results.

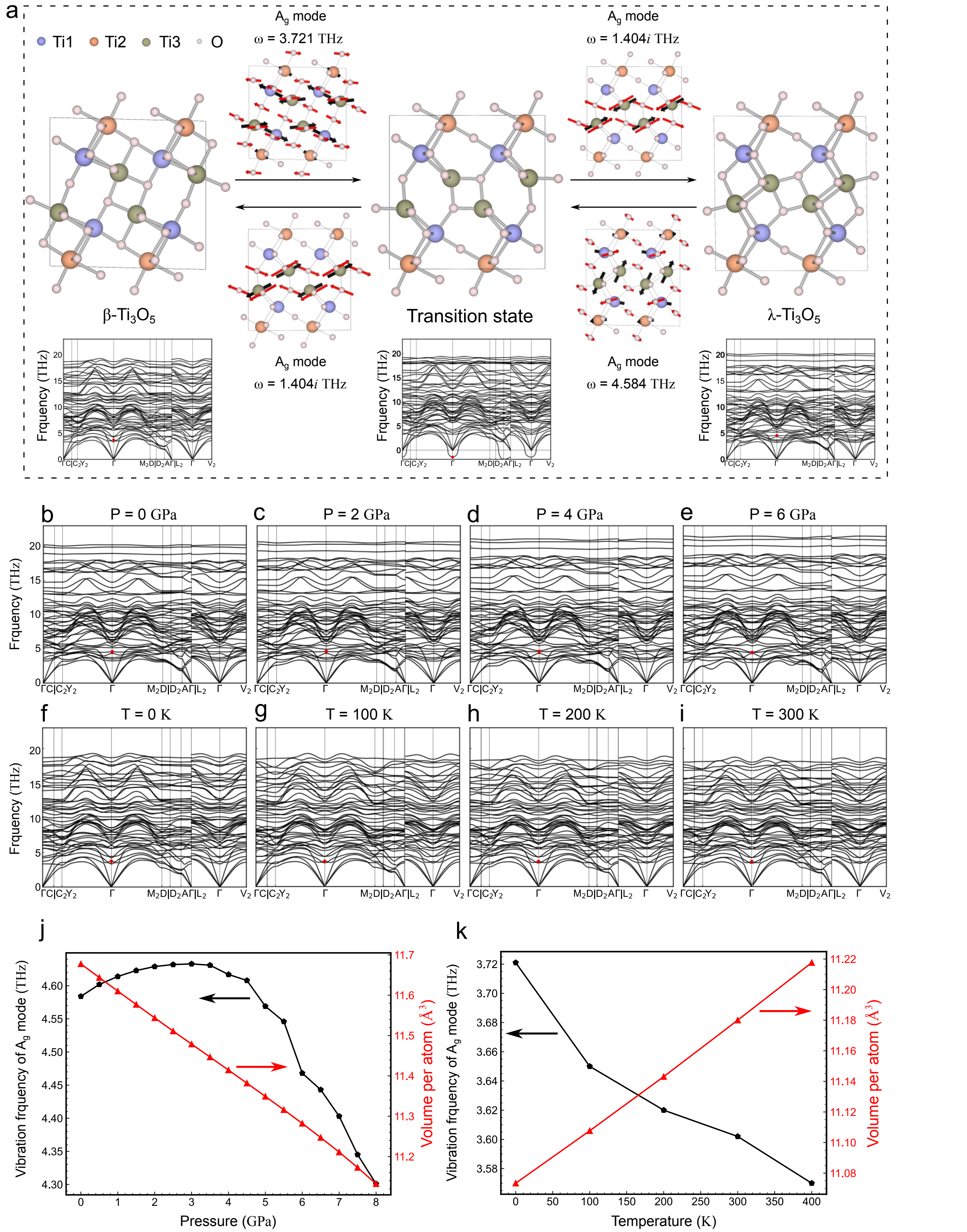

Supplementary Fig. 3. Phonon mode analysis. **a**, The decisive phonon modes that drive the phase transformation from β to λ, and vice versa. The arrows on the atoms indicate the atomic displacements associated with the specified phonon mode. **b-e**, Pressure-dependent phonon dispersions of λ-Ti_3O_5. **f-i**, Temperature-dependent phonon dispersions of β-Ti_3O_5. **j**, Pressure-dependent A_g phonon mode frequencies and system volumes of λ-Ti_3O_5. **k**, Temperature-dependent A_g phonon mode frequencies and system volumes of β-Ti_3O_5. Note that the red dots in the plots of phonon dispersions indicate the A_g phonon mode.

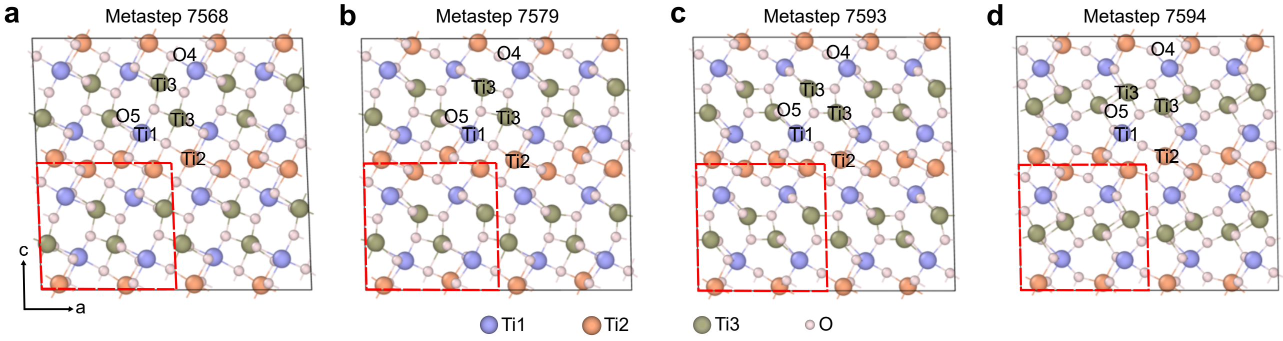

Supplementary Fig. 4. Snapshots extracted from a metadynamics simulation at 500 K. **a**, Snapshot extracted at metastep 7568. **b**, Snapshot extracted at metastep 7579. **c**, Snapshot extracted at metastep 7593. **d**, Snapshot extracted at metastep 7594. For a better visualization, here the employed supercell (indicated by red dashed lines) is doubled along both the *a* and *c* directions.

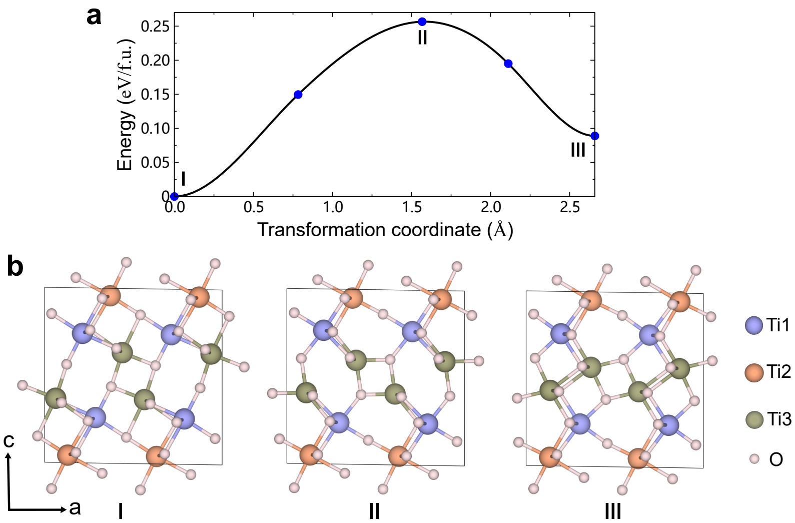

Supplementary Fig. 5. Variable-cell climbing image nudged elastic band calculations using DFT. a, The relative energy as a function of transformation coordinate. The energy is given in electron volt per formula unit (eV/f.u.). The calculated data are represented by blue circles, while the black curve corresponds to the spline fit. **b**, The structures of initial (I, corresponding to β), transitional (II), and final (III, corresponding to λ) phases.

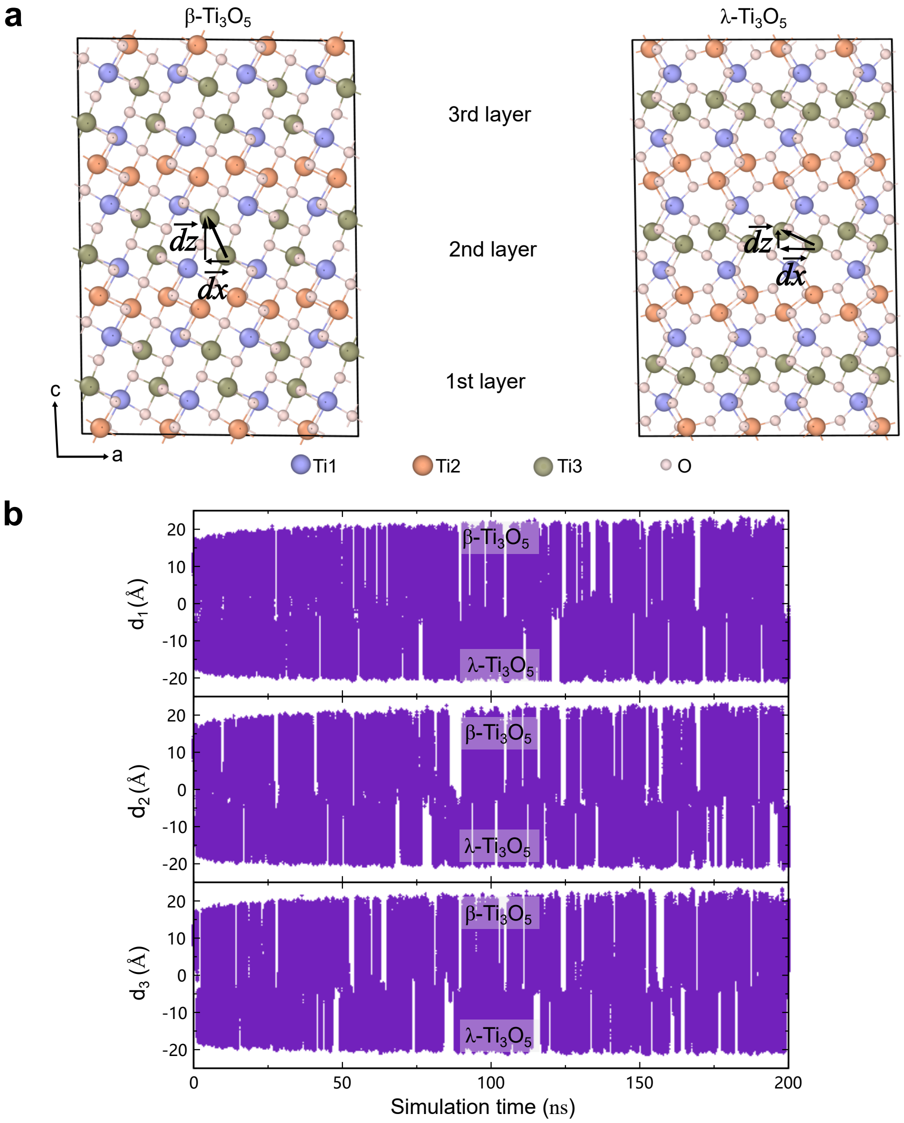

Supplementary Fig. 6. Collective variables design and their time evolution. a, The collective variable employed for describing the layer-by-layer growth in a three-layer structure are defined as $d_1 = \sum_{i\in\text{1st Ti3}-\text{Ti3 layer}} \left([\vec{dx}]^i + [\vec{dz}]^i\right)$, $d_2 = \sum_{i\in\text{2nd Ti3}-\text{Ti3 layer}} \left([\vec{dx}]^i + [\vec{dz}]^i\right)$, and $d_3 = \sum_{i\in\text{3rd Ti3}-\text{Ti3 layer}} \left([\vec{dx}]^i + [\vec{dz}]^i\right)$. Here, $[\vec{dx}]^i$ and $[\vec{dz}]^i$ represent the projected displacements of the i-th Ti3-Ti3 dimer in a layer along the a and c axes, respectively. **b**, The collective variables as a function of simulation time. The metadynamics simulations here were performed at 600 K and 0 GPa using a supercell of 384 atoms containing there Ti3-Ti3 layers along the c direction.

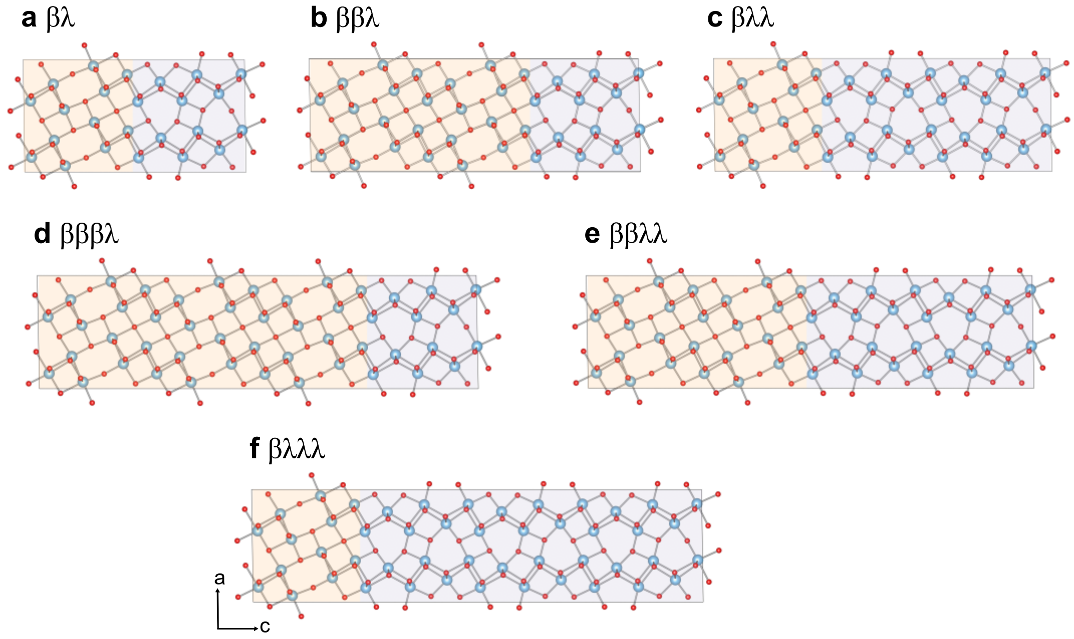

Supplementary Fig. 7. Predicted novel metastable phases. **a**, Structure of $\beta\lambda$-stacking phase. **b**, Structure of $\beta\beta\lambda$-stacking phase. **c**, Structure of $\beta\lambda\lambda$-stacking phase. **d**, Structure of $\beta\beta\beta\lambda$-stacking phase. **e**, Structure of $\beta\beta\lambda\lambda$-stacking phase. **f**, Structure of $\beta\lambda\lambda\lambda$-stacking phase. The light yellow and purple colors represent the β-like and λ-like local structural motifs, respectively. As an example, the notation of "$\beta\beta\lambda$" indicates a metastable phase formed by sequentially stacking the β-like, β-like and λ-like local structural motifs along the c direction. We note that all structures with any combination of β-like and λ-like structural motifs along the c axis are dynamically stable, but here only the metastable phases with the number of stacking layers up to four are shown for brevity. The large blue and small red balls represent Ti and O atoms, respectively.

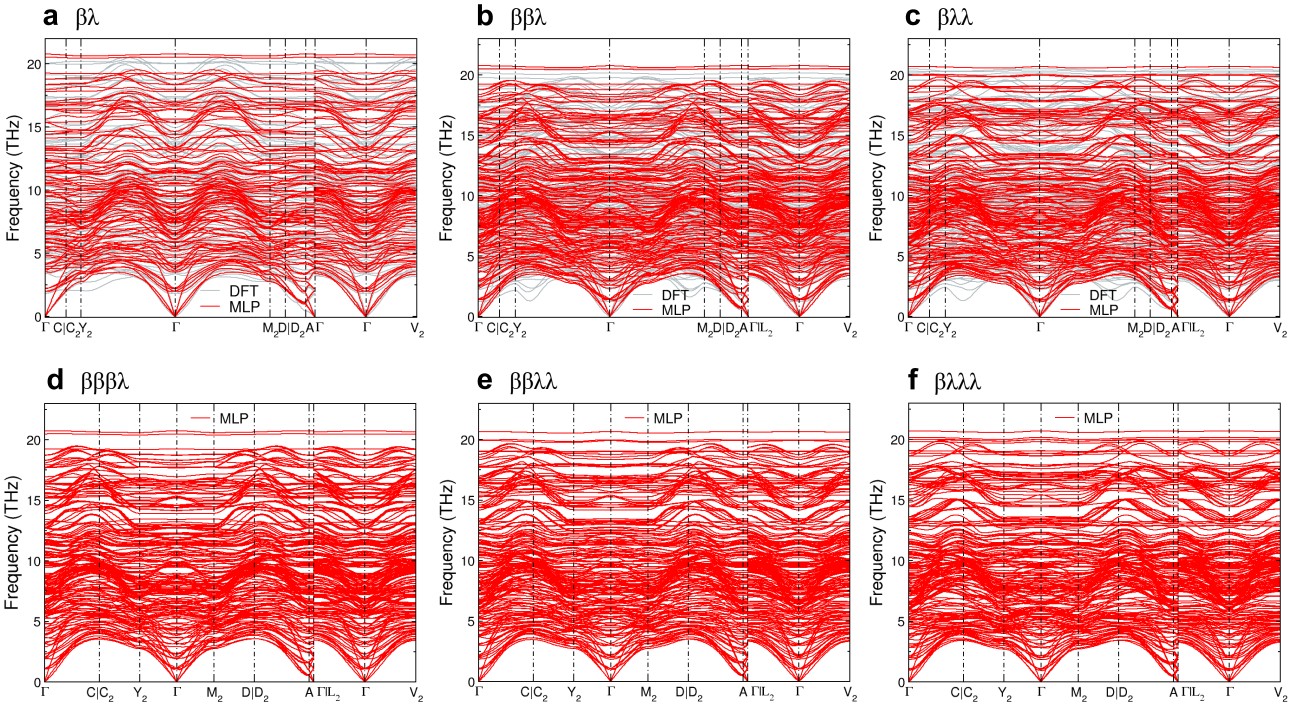

Supplementary Fig. 8. Machine learning potential (MLP) predicted phonon dispersion relationships for the metastable phases in Supplementary Fig. 7. **a**, Phonon dispersion of $\beta\lambda$-stacking phase. **b**, Phonon dispersion of $\beta\beta\lambda$-stacking phase. **c**, Phonon dispersion of $\beta\lambda\lambda$-stacking phase. **d**, Phonon dispersion of $\beta\beta\beta\lambda$-stacking phase. **e**, Phonon dispersion of $\beta\beta\lambda\lambda$-stacking phase. **f**, Phonon dispersion of $\beta\lambda\lambda\lambda$-stacking phase. For the phases ($\beta\lambda$, $\beta\beta\lambda$, and $\beta\lambda\lambda$) with small supercells, the DFT computed ones (in gray) are also shown for comparison.

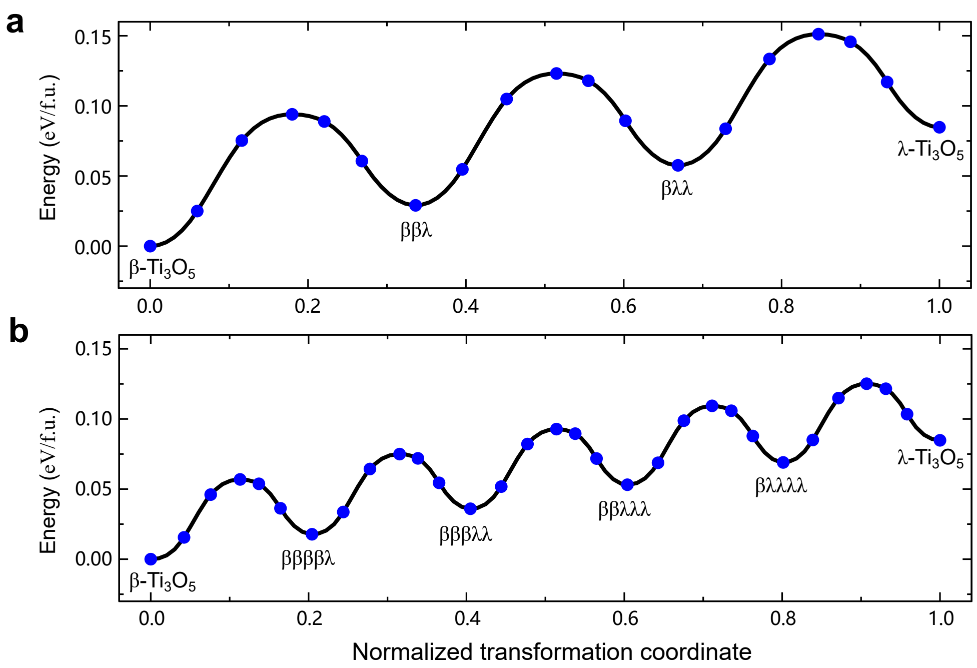

Supplementary Fig. 9. Variable-cell climbing image nudged elastic band calculations using machine learning potential. **a**, The relative energy as a function of normalized transformation coordinate for a three-layer-stacking supercell including 48 atoms. **b**, The relative energy as a function of normalized transformation coordinate for a five-layer-stacking supercell including 80 atoms. The energy is given in electron volt per formula unit (eV/f.u.).

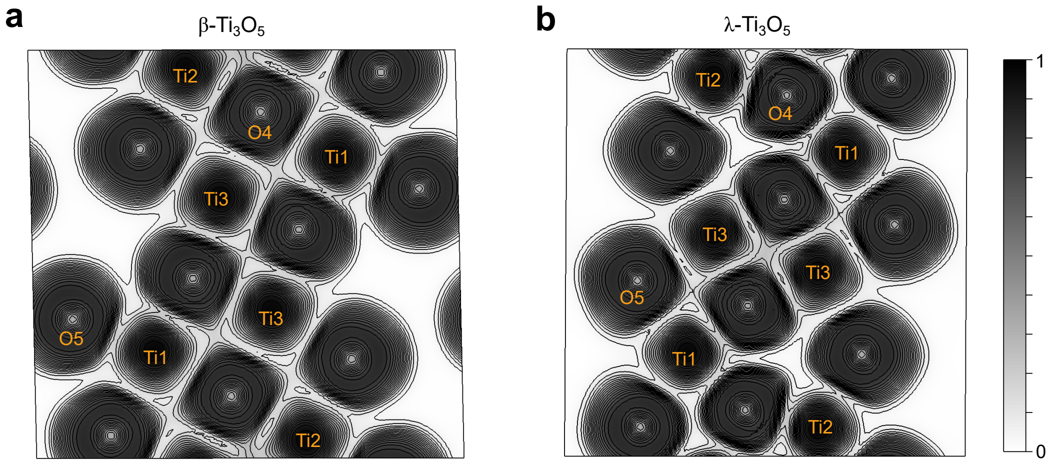

Supplementary Fig. 10. DFT calculated electron localization functions. **a**, β-Ti_3O_5. **b**, λ-Ti_3O_5. The relatively weak Ti3-O4 and Ti3-O5 chemical bonds can be identified for β-Ti_3O_5 and λ-Ti_3O_5, respectively.

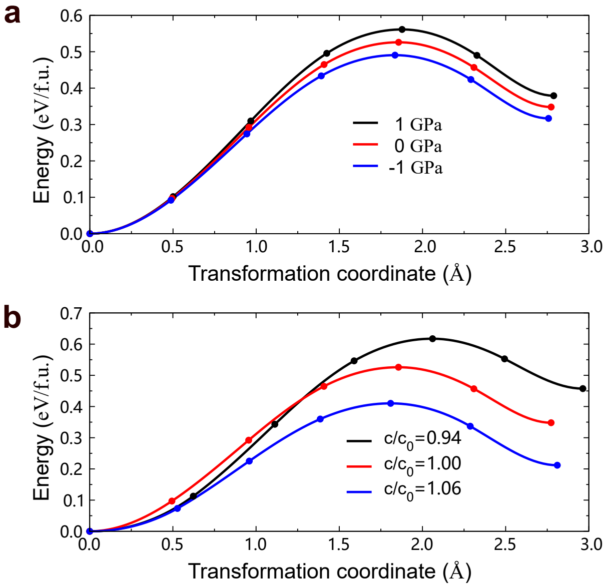

Supplementary Fig. 11. Pressure and strain dependence of energy barrier for the β to λ transition. **a**, Pressure dependence of energy barrier. **b**, *c*-axis unidirectional tensile strain dependence of energy barrier. Note that here the energy barriers were computed by fixed-cell climbing image nudged elastic band calculations using machine learning potential. The energy is given in electron volt per formula unit (eV/f.u.).

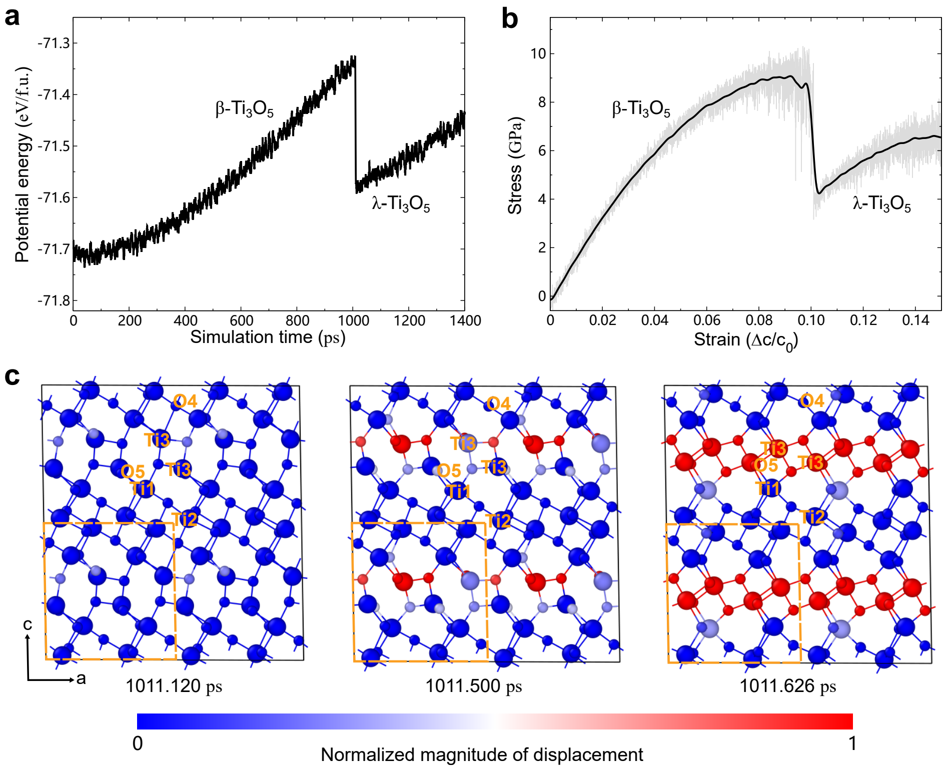

Supplementary Fig. 12. Direct molecular dynamics simulations under *c*-axis unidirectional continuous tensile strain (strain rate 10^8/s) at 300 K and 0 GPa using a 96-atom cell. **a**, Evolution of potential energy with respect to simulation time. The energy is given in electron volt per formula unit (eV/f.u.). **b**, Stress-strain curve. **c**, Snapshots close to the phase transition. For a better visualization, here the employed supercell (indicated by yellow dashed lines) is doubled along both the *a* and *c* directions.

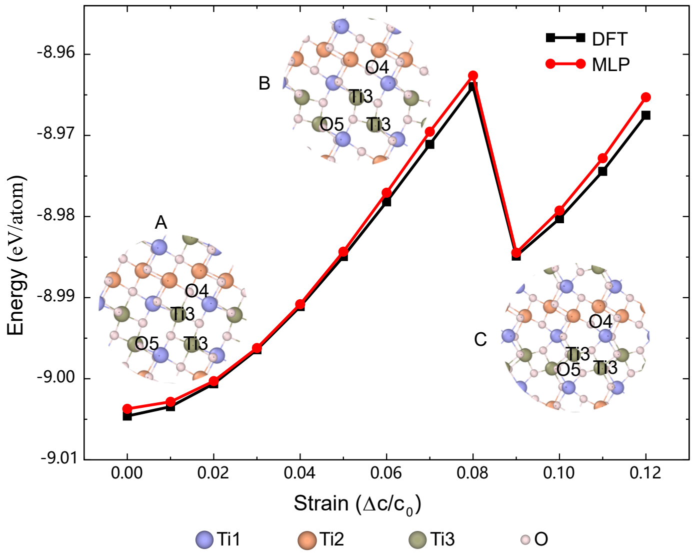

Supplementary Fig. 13. Total energies of a 32-atom cell predicted by DFT and machine learning potential (MLP) as a function of tensile strain along the *c* axis. The structures indicated by A, B, and C correspond to the strains ($\Delta c/c_0$) of 0.0, 0.08 and 0.09, respectively. c_0 is the c-axis lattice constant of β-Ti_3O_5.

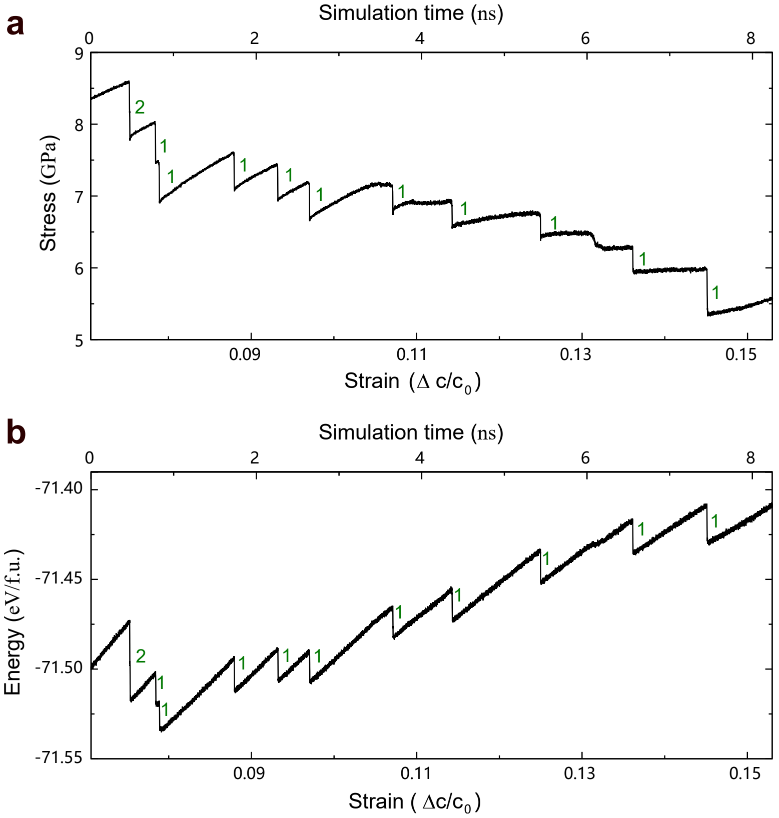

Supplementary Fig. 14. Evolution of the stress and potential energy of a large cell including 165,888 atoms with respect to strain under *c*-axis unidirectional continuous tensile strain. a, Evolution of stress. **b**, Evolution of potential energy. The employed strain rate here is 10^6/s. The values close to each jump indicate the number of layers undergoing the β to λ transition. The energy is given in electron volt per formula unit (eV/f.u.).

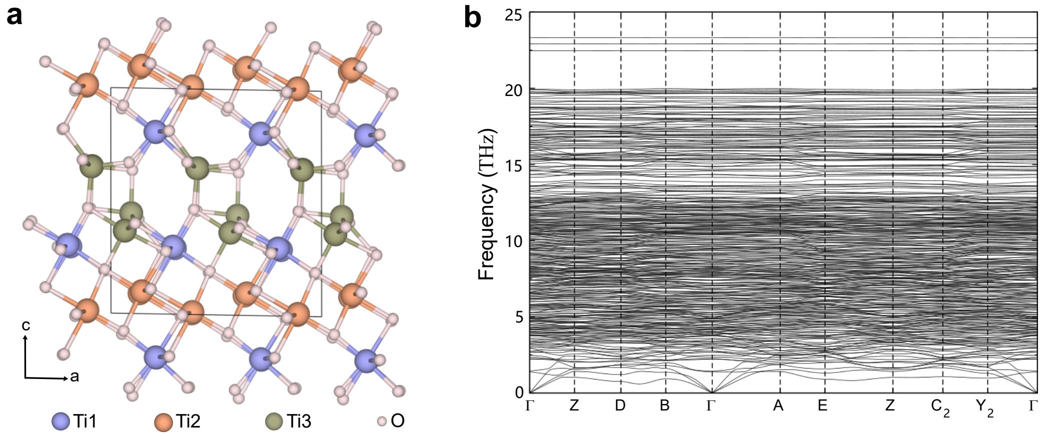

Supplementary Fig. 15. Defect-like metastable phase and its phonon dispersion. **a**, Crystal structure of the defect-like metastable phase. **b**, Phonon dispersion of the defect-like metastable phase. The metastable phase was obtained by elongating the *c* lattice constant in terms of the stable β-Ti_3O_5 phase and fully relaxing the other degree of freedoms. It will transform to the β phase after full structural relaxation (i.e., constraints are eliminated). The detailed structure information of this metastable phase is provided in Supplementary Data 1. The large and small balls in **a** represent the Ti and O atoms, respectively.

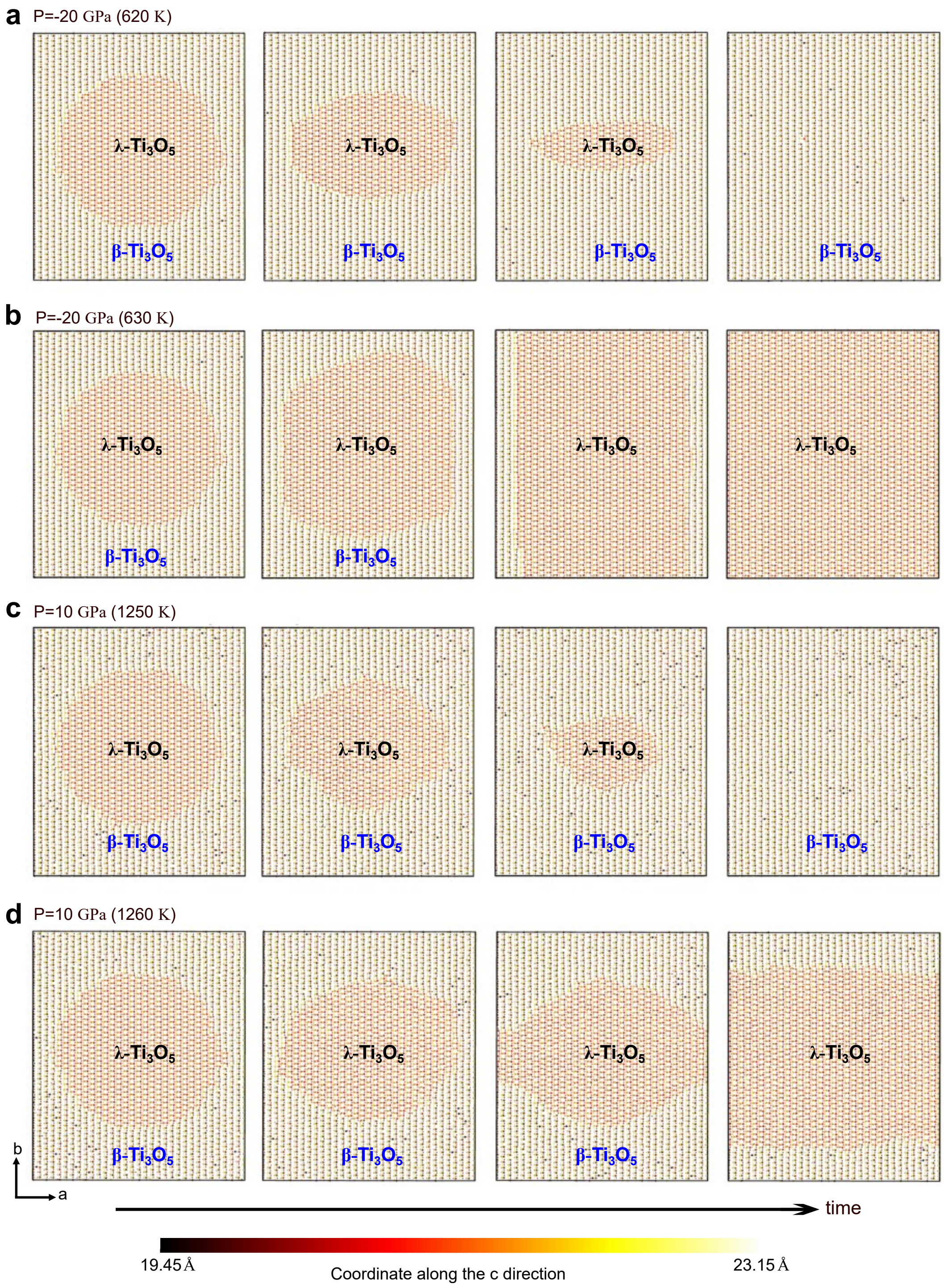

Supplementary Fig. 16. Large-scale in-plane phase growth molecular dynamics simulations at different temperatures and pressures. **a**, At the pressure of −20 GPa and the temperature of 620 K. **b**, At the pressure of −20 GPa and the temperature of 630 K. **c**, At the pressure of 10 GPa and the temperature of 1250 K. **c**, At the pressure of 10 GPa and the temperature of 1260 K.

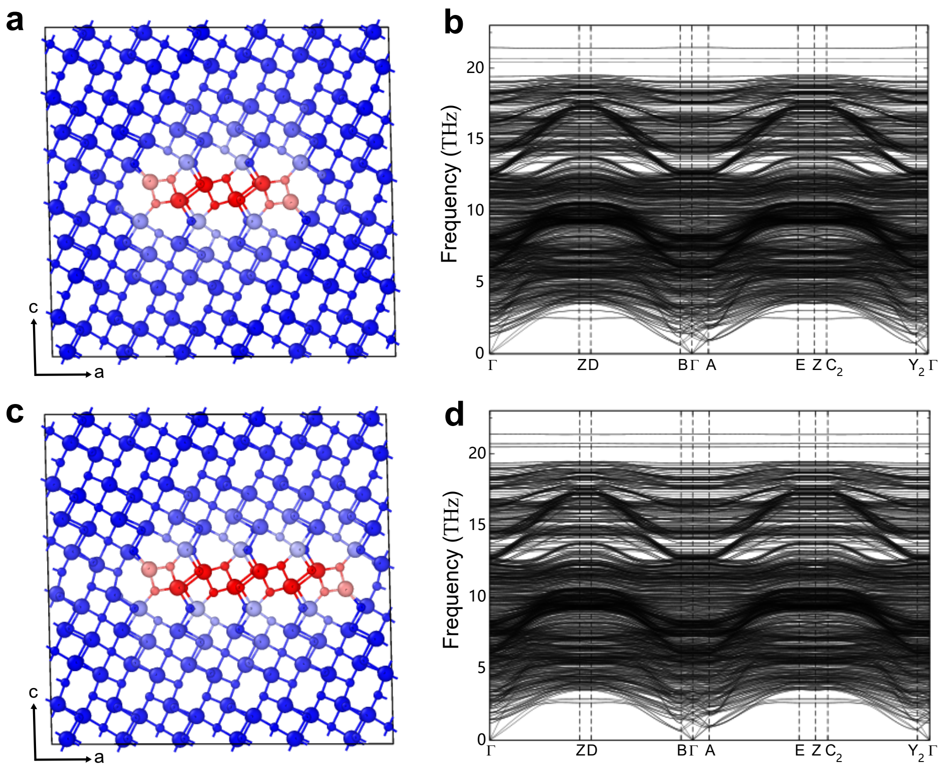

Supplementary Fig. 17. Metastable phases observed during the in-plane growth process and their phonon dispersions. a-b Crystal structure of metastable phase B2 (Fig. 5 of the main text) and its phonon dispersion. **c-d** Crystal structure of metastable phase B4 (Fig. 5 of the main text) and its phonon dispersion. The large and small balls in **a** and **c** represent the Ti and O atoms, respectively.

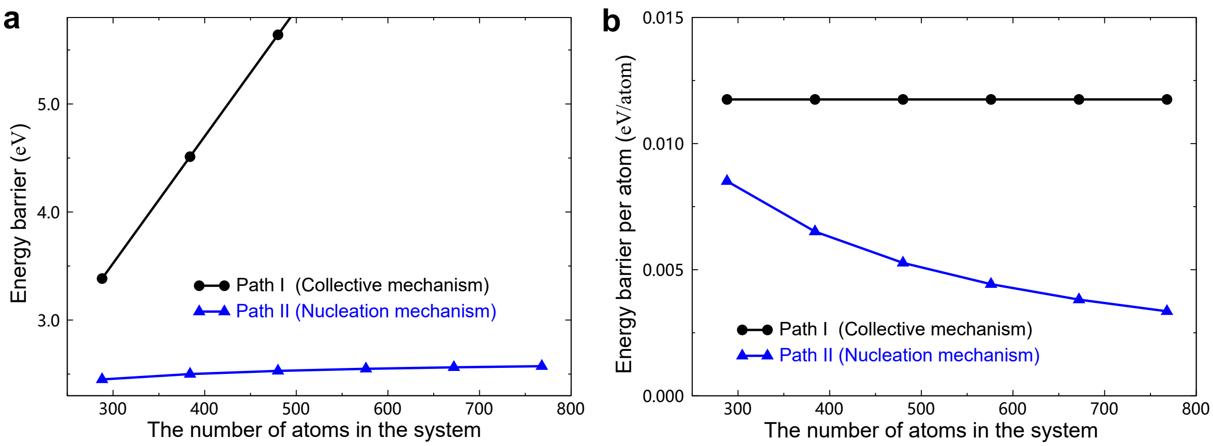

Supplementary Fig. 18. System size dependence of energy barrier. a, Calculated energy barrier as a function of system size for two different phase transformation mechanisms from β to λ phases, i.e., collective mechanism (Path I) and nucleation mechanism (Path II). Note that for a better presentation the results for the case of the collective mechanism beyond the system size of 500 atoms are not shown. **b**, Calculated ratio between the energy barrier and the system size as a function of system size.

Supplementary Tables

Supplementary Table 1. The lattice parameters, volumes, energy differences between the two phases ΔE, phase transition temperature T_c, and phase transition enthalpy ΔH for a 32-atom unit cell predicted by PBE-derived machine learning potential (MLP), PBE, PBE+U (U=4.4 eV), PBEsol, SCAN, and r^{2}SCAN+D3. Note that nonmagnetic setups are adopted for all the methods except for PBE+U and r^{2}SCAN+D3 which consider the magnetic degrees of freedom. NM-M, AFM-I, FM-M, NM-S, and PM-M denote the nonmagnetic metal, antiferromagnetic insulator, ferromagnetic metal, nonmagnetic semiconductor, and paramagnetic metal, respectively. The energy is given in electron volt per formula unit (eV/f.u.).

	PBE-MLP	PBE	PBE+D3	PBE+U	PBEsol	SCAN	r^{2}SCAN+D3	Experiments
β-Ti_3O_5 ($C2/m$)	This work	This work	This work	This work	This work	This work	ref. [1]	ref. [2]
a (Å)	9.824	9.809	9.685	10.243	9.635	9.769	9.697	9.753
b (Å)	3.852	3.857	3.842	3.913	3.841	3.804	3.850	3.800
c (Å)	9.368	9.361	9.287	9.698	9.219	9.330	9.277	9.444
β (°)	91.333	91.204	91.024	92.321	91.089	91.355	91.068	91.532
Volume (Å^3)	354.35	354.10	345.50	388.35	341.09	346.66	346.30	349.90
Electronic state	—	NM-M	NM-M	AFM-I	NM-M	NM-M	FM-M	NM-S
λ-Ti_3O_5 ($C2/m$)	This work	This work	This work	This work	This work	This work	ref. [1]	ref. [2]
a (Å)	9.840	9.851	9.699	10.114	9.568	9.655	9.808	9.831
b (Å)	3.795	3.787	3.801	3.881	3.815	3.799	3.808	3.788
c (Å)	10.007	10.023	9.908	10.320	9.809	9.928	9.930	9.970
β (°)	90.775	90.810	90.234	90.656	92.679	90.595	91.214	91.291
Volume (Å^3)	373.68	373.90	365.21	405.02	357.64	364.09	370.82	371.21
$\Delta E(\lambda-\beta)$ (eV/f.u.)	0.085	0.078	0.212	−0.175	0.174	0.163	0.110	—
$T_c(\lambda-\beta)$ (K)	535	—	—	—	—	—	525	470
$\Delta H(\lambda-\beta)$ (eV/f.u.)	0.091	—	—	—	—	—	0.098	0.124± 0.01
Electronic state	—	NM-M	NM-M	AFM-I	NM-M	NM-M	FM-M	PM-M
α-Ti_3O_5 ($Cmcm$)	This work	This work	This work	This work	This work	This work	This work	ref. [3]
a (Å)	3.789	3.780	3.773	3.878	3.777	3.748	3.768	3.798
b (Å)	9.885	9.916	9.813	10.185	9.764	9.842	9.778	9.846
c (Å)	9.995	9.970	9.885	10.201	9.805	9.920	9.866	9.988
Volume (Å^3)	374.33	373.73	366.00	402.95	361.64	365.89	363.50	373.50
$\Delta E(\alpha-\beta)$ (eV/f.u.)	0.100	0.118	0.244	−0.301	0.239	0.216	0.258	—
Electronic state	—	NM-M	NM-M	AFM-I	NM-M	NM-M	NM-M	—

Supplementary Notes

Supplementary Note 1: The choice of the density functional

As mentioned in the main text, in this work the standard PBE functional with nonmagnetic setups were adopted for all the first-principle calculations. This is a very reasonable choice. The reasons are given as follows:

(*i*) The density functional theory calculations employing nonmagnetic setups yield good descriptions of the electronic structures of both β and λ phases, e.g., the formation of a bipolaron (with no spin) of Ti3-Ti3 caused by σ-type bonding of d_{xy} orbitals of Ti3 atoms in the β-Ti_3O_5 phase and the formation of slipped π-stacking between the d_{xy} orbital on Ti2 and the d_{xy} orbital on Ti3 in the λ-Ti_3O_5 phase [4–6].

(*ii*) The density functionals considering the magnetic setups often predict incorrect ground states that are not consistent with experiments. For instance, the hybrid functional M06-D3 predicts the β-Ti_3O_5 phase to be an antiferromagnetic (AFM) semiconductor, while predicts the λ-Ti_3O_5 phase to be a ferromagentic (FM) semiconductor [1]. The r^2SCAN-D3 method yields ferromagentic metals for both β- and λ-Ti_3O_5 phases [1], while the PBE+U method predicts that the AFM order was found to be the ground states for both phases [7]. With U=4.4 eV the PBE+U method predicts an AFM insulator for all the three phases (β, λ, and α) and even yield qualitatively wrong negative values for the energy difference between the two phases (see Supplementary Table 1). We recall that in experiment the β phase is a nonmagnetic semiconductor, while the λ phase is a weak Pauli paramagnetic metal [4,5].

(*iii*) The PBE functional with the nonmagnetic setups gives an excellent description of the experimental lattice parameters of β, λ as well as α phases as compared to other density functionals such as PBEsol and SCAN (see Supplementary Table 1).

(*iv*) Considering the magnetic degrees of freedom hardly changes the thermodynamical properties of both phases [1]. On the one hand, the calculated energy differences between different spin configurations are found to be small (see Table 1 of ref. [1]). On the other hand, the activation barriers from β to λ only change slightly between different magnetic configurations (see Table 3 of ref. [1]). We also compared the phase transition temperature T_c and phase transition enthalpy ΔH predicted by our PBE-derived machine learning potential (MLP) to the results predicted by r^2SCAN+D3 as well as the experimental data. It can be seen from Supplementary Table 1 that the MLP fully accounting for the anharmonic phonon-phonon interactions yields a T_c of 535 K and ΔH of 0.091 eV/f.u., in good agreement with the predictions by the r^2SCAN+D3 method within the simpler harmonic approximation (T_c=525 K and ΔH=0.098 eV/f.u.) [1]. Both are in line with the experimental results (T_c=460 K and ΔH=0.124$\pm$0.01 eV/f.u.) [2]. However, PBE is computationally cheaper and needs a faction of the computational cost of the meta-GGA r^2SCAN functional. Furthermore, including the magnetic degree of freedom will dramatically increase the complexity of MLP model. As far as we know, only few works currently attempt at incorporating the spin descriptor besides the structural descriptor into the MLP model.

Finally, we would like to briefly discuss the effect of the D3 dispersion correction on the structural and thermodynamical properties of β- and λ-Ti_3O_5. Generally, the D3 method is not accurate for ionic materials: in Ti_3O_5 charge is transferred from the Ti atoms to the oxygen atoms, but D3 and many other *posterior* corrections have not been designed to take this charge transfer into account. Furthermore, the D3 corrections have been parameterized to describe interactions through the vacuum, whereas in bulk materials the dispersion corrections are strongly screened by the surrounding atoms. The minimum level of complexity are thus many-body-dispersion corrections that account for the interplay between the polarizable entities. Hence, there is no reason to believe that D3 captures the relevant physics in Ti_3O_5. Indeed, we found that although both PBE and PBE+D3 give a correct positive energy difference between the two phases, PBE+D3 yields a significantly large value of 0.212 eV/f.u., far larger than the experimentally measured phase transition enthalpy (0.124$\pm$0.01 eV/f.u.) [2] (see Supplementary Table 1). In addition, we found that inclusion of the D3 correction slightly deteriorates the description of lattice parameters (see Supplementary Table 1). By contrast, the standard PBE functional yields excellent structural and thermodynamical properties of both phases. The predicted thermodynamical temperature-pressure phase diagram using the PBE-derived MLP is in good agreement with experiment (see Fig. 1 of the main text). We would like to stress that although the choice of the density functional would to some extent quantitatively change the actual values of the phase transition temperature and pressure, our main discovery of unusual layer-by-layer phase transformation initiated by kinetically favorable in-plane nucleated mechanism will remain unchanged by the change of the density functional.

Supplementary references

[1] Jütten, S. & Bredow, T. First-principles investigation of electronic properties and phase transition of Ti_3O_5. *The Journal of Physical Chemistry C* **126**, 7809–7817 (2022).

[2] Tokoro, H. *et al.* External stimulation-controllable heat-storage ceramics. *Nature Communications* **6**, 7037 (2015).

[3] Onoda, M. Phase transitions of Ti_3O_5. *Journal of Solid State Chemistry* **136**, 67–73 (1998).

[4] Ohkoshi, S.-i. *et al.* Synthesis of a metal oxide with a room-temperature photoreversible phase transition. *Nature Chemistry* **2**, 539–545 (2010).

[5] Kobayashi, K. *et al.* Electronic structure and correlation in β-Ti_3O_5 and λ-Ti_3O_5 studied by hard x-ray photoelectron spectroscopy. *Phys. Rev. B* **95**, 085133 (2017).

[6] Yang, B. *et al.* Flatband λ-Ti_3O_5 towards extraordinary solar steam generation. *Nature* **622**, 499 (2023).

[7] Mariette, C. *et al.* Strain wave pathway to semiconductor-to-metal transition revealed by time-resolved X-ray powder diffraction. *Nature Communications* **12**, 1239 (2021).
