# Supplementary material for: Layer-by-layer phase transformation in Ti3O5 revealed by machine-learning molecular dynamics simulations
Source: Nat Commun. 2024 Apr 9;15:3079. doi: 10.1038/s41467-024-47422-1 (PMC11004112; doi:10.1038/s41467-024-47422-1)
Supplement: Supplementary file 3 — Description of Additional Supplementary Files [file 41467_2024_47422_MOESM3_ESM.pdf]

Description of Additional Supplementary Files:

Supplementary Data 1: Structure models employed in this work.

Supplementary Movie 1: Large-scale molecular dynamics simulations under tensile strain at 300 K and 0 GPa.

Supplementary Movie 2: Large-scale twodimensional phase growth molecular dynamics simulations at 1070 K and 1080 K under a pressure of 0 GPa..

Supplementary Movie 3: Variable-cell climbing image nudged elastic band calculations for transforming one layer of βTi_3O_5 from β-like to λ-like structural motifs.
